# Effect of fermented garlic extract containing nitric oxide on radial artery pulse waves in hypertension patients: a feasibility observational study

**DOI:** 10.3389/fnut.2025.1433623

**Published:** 2025-04-22

**Authors:** Jihye Kim, Gyeong cheul Kim, HeeJung Kang, Youngju Jeon

**Affiliations:** ^1^Digital Health Research Division, Korea Institute of Oriental Medicine, Daejeon, Republic of Korea; ^2^Department of Diagnostics, College of Korean Medicine, Dong-Eui University, Busan, Republic of Korea; ^3^The Medical Research Institute of Hot Spring, Dong-Eui University, Busan, Republic of Korea; ^4^Department of Korean Medicine, Graduate School, Dong-Eui University, Busan, Republic of Korea; ^5^DAEYOMEDI Co., Ltd., Ansan-si, Republic of Korea

**Keywords:** nitric oxide, fermented garlic extract, pulse wave, radial artery, blood pressure, artery stiffness, hypertension, noninvasive radial artery tonometry device

## Abstract

**Background:**

This study aimed to evaluate the effect of fermented garlic extract (FGE) containing nitric oxide (NO) on arterial pulse waves in hypertension patients using a noninvasive radial artery tonometry device.

**Materials and methods:**

Forty-one participants were recruited for this study investigating changes in arterial pulse wave characteristics following the ingestion of FGE containing NO over a 2-week period. Arterial pulse wave measurements were taken before and 15, 20, and 25 min after FGE administration and 2 weeks after the end of the ingestion period.

**Results:**

One participant withdrew, and five participants refused to undergo pulse wave measurements. These six participants were excluded, resulting in 35 participants being included for analysis. Fifteen minutes after the administration of FGE with NO, the systolic and diastolic blood pressure (BP) significantly decreased. The radial augmentation index (RAI), width (w), width/time (w/t) ratio, and stroke volume index (SVI) significantly decreased, while the mean pulse width significantly increased. Notably, the RAI, w, w/t ratio, and SVI exhibited a decreasing trend at 15, 20, and 25 min compared to the values before the administration of FGE. After 2 weeks of ingestion, no pulse wave variables showed significant changes compared to those before the administration of FGE.

**Conclusion:**

The oral administration of low-dose FGE containing NO showed acute positive effects on the wrist artery, including a reduction in BP and an improvement in arterial stiffness. These findings suggest that this study successfully evaluated the effects of FGE containing NO using quantitative and objective pulse parameters as noninvasive indicators.

## 1 Introduction

Hypertension is a significant risk factor for cardiovascular and kidney diseases and an increasing public health concern worldwide (1). Defined as a systolic blood pressure (BP) ≥ 140 mmHg and/or a diastolic BP ≥ 90 mmHg, hypertension has a prevalence of approximately one-quarter (25%) of the adult population (2).

Endothelial dysfunction is the first step in the development of atherosclerotic disease; it is characterized by impaired bioavailability of nitric oxide (NO), and it occurs early among all known risk factors for both hypertension and cardiovascular disease (3).

Individuals with hypertension often exhibit impaired NO bioactivity. NO, a crucial signaling molecule, plays a pivotal role in blood vessel health and the regulation of BP (3), and impaired NO bioactivity has emerged as a major aspect of hypertension. Compared with control mice, mice with disrupted endothelial NO synthase genes exhibited elevated BP, indicating a genetic link between impaired NO bioactivity and hypertension (4, 5). Clinical studies have shown that hypertension patients exhibit a blunted arterial vasodilatory response to endothelium-dependent vasodilator infusion, and inhibition of NO elevates BP. Impaired NO bioactivity is also implicated in arterial stiffness, a major mechanism of systolic hypertension (3, 6, 7). Elucidating the mechanisms underlying impaired NO bioactivity in hypertension could have significant implications for its treatment.

Garlic has been traditionally employed across various cultures for its potential health benefits. Relevant to hypertension, some studies have suggested that garlic may positively influence BP levels. Many of the therapeutic actions of garlic parallel the physiological effects of NO and may be explained by its ability to increase intracellular NO synthase activity (8). The benefits of garlic in hypertension may be due to its potential to alter markers related to the function and health of the vascular endothelium. Specifically, oxidative stress-induced damage to endothelial cells can hinder their NO production, leading to endothelial dysfunction. Several garlic formulations exhibit considerable antioxidant properties (9), indicating that garlic consumption may mitigate oxidative harm to endothelial cells and enhance vascular function (10).

Technological modifications of different forms of raw garlic, such as smoked garlic, garlic oil macerate, garlic juice, garlic powder, and fermented garlic, have been developed to alleviate discomfort and increase dietary tolerance (11). Fermented garlic extract (FGE) enriched with NO has emerged as a potential therapeutic agent due to its vasodilatory properties and purported cardiovascular benefits (12). Understanding the impact of FGE on arterial function is essential for elucidating its therapeutic potential in hypertension patients.

This study aimed to evaluate the effects of FGE containing NO on pulse wave dynamics in hypertensive patients using a noninvasive radial artery tonometry device. We hypothesized that the consumption of FGE would alter radial artery pulse wave patterns in a manner similar to the pulse wave features observed in the upper arm. To the best of our knowledge, this is the first study to provide preliminary evidence on the effects of FGE containing NO on radial artery pulse wave dynamics, whereas previous studies have predominantly focused on cerebral blood flow and blood pressure measured at the upper arm. The findings of this study may enhance our understanding of FGE as a potential therapeutic intervention for hypertension.

## 2 Materials and methods

### 2.1 Hypothesis

The hypothesis of this study is that 2 weeks of ingestion of FGE containing NO will improve cardiac vascular system parameters, including BP and arterial stiffness.

### 2.2 Ethics approval

The Institutional Review Board of Dong-Eui University approved the study protocol (Institutional Review Board approval number: DIRB-202210-HR-E-34). This study was registered on the Korean National Clinical Trial Registry (Cris.nih.go.kr identifier: KCT0008234).

### 2.3 Study design

This feasibility clinical study was conducted as a prospective, interventional, single-arm study with a 2-week intervention period. The study was conducted from December 2022 to January 2023 at Dong-Eui University in the Republic of Korea. Table 1 outlines the study timeline, including enrollment, measurements, and data collection. Data collected included general characteristics (age, education, occupation, income, smoking status, alcohol consumption, physical activity, disease history, and medication use), as well as anthropometric measurements and vital signs.

**Table 1:** Details of the study timeline, including enrollment, measurements, and data collection.

	Visit 1	Visit 2				Visit 3
	Enrollment	Measurement				Close-out
Timepoint	Screening	Pre	After 15 min	After 20 min	After 25 min	Post
Enrollment						
Informed consent	x					
Inclusion/exclusion criteria	x					
Sociodemographic profile	x					
Medical history	x					
Vital signs	x	x				x
Intervention						
FGE dispensed		x				
FGE compliance checked						x
Assessment						
Change in medical history		x				x
Blood pressure	x	x				x
Noninvasive radial artery tonometry		x	x	x	x	x
Blood circulation questionnaire		x				x
Safety assessment		x	x	x	x	x

All participants were instructed to take the FGE containing NO orally four times a day: three doses after meals (at 8 a.m., 1 p.m., and 8 p.m.) and one dose before bedtime. Each dose consisted of 60 ml, resulting in a total daily intake of 240 ml. The ingestion period lasted for 2 weeks. Compliance with taking the FGE was assessed by tracking the return of unused FGE and reviewing self-reported adherence logs. The compliance rate was calculated as the percentage of prescribed doses consumed by participants over the two-week study period. Participants were contacted regularly to reinforce adherence and address any barriers to compliance. Additionally, reminders were provided to ensure participants followed the intervention protocol.

All participants underwent a health check to assess their disease history according to the inclusion and exclusion criteria. Participants with structural valve abnormalities, an artificial heart or valve, a pacemaker, those undergoing dialysis, or with rectangular veins were excluded from the study.

The inclusion criteria were as follows: (1) residents of Busan city or Gyungnam province for over 20 years; (2) patients of both sexes; and (3) patients over 30 years of age who had hypertension or an SBP greater than 130 mmHg.

The exclusion criteria were as follows: (1) vascular abnormality in the radial area due to the puncture; (2) serious shaking of the hand; (3) difficulty maintaining the measurement posture due to paralysis in the wrist area; (4) structural valve abnormalities; (5) artificial heart, artificial valve, or pacemaker; (6) dialysis; (7) serious arrythmia; (8) no pulse measured by the research device; (9) pregnancy or lactation; (10) heart rate less than 40 beats/min or more than 183 beats/min; (11) participation in other clinical studies within 1 month (30 days) before the start of the clinical study; and (12) other principal investigators or researchers judging that inclusion in the study was inappropriate.

All the participants provided informed consent for participation in the study. This study was conducted in accordance with the principles of the Declaration of Helsinki 1975 (27) and complied with Good Clinical Practice guidelines. An independent board monitored patient safety throughout the study.

### 2.4 Fermented garlic extract containing nitric oxide

The FGE consists of fermented garlic and water and contains NO. The FGE used in this study, named Root of Hundred Years, was prepared by RIVER OF LIFE Co. (Nonsan-si, Chungcheongnam-do, Republic of Korea). This FGE was approved by the Ministry of Food and Drug Safety (product license number and date: 2018047043367, issued on 12/02/2019). The NO and 126 superoxide dismutase activity of the FGE are summarized in Table 2.

**Table 2:** Nitric oxide and superoxide dismutase activity of FGE.

Sample dilution ratio	SOD activity (inhibition rate, %)
1/5^0^	76.43 ± 0.70
1/5^1^	52.3 ± 2.54
1/5^2^	24.57 ± 5.96
1/5^3^	7.63 ± 1.84
IC50	1/5.26
SOD activity (U/ml)	263
NO (nmol/L)	5783037.48 ± 68325.48

SOD, superoxide dismutase; NO, nitric oxide.

The manufacturing process began with the removal of the outer skin from raw garlic, followed by thorough washing, sterilization, and grinding. The resulting mixture was then diluted with water at a ratio of 1:9 (weight/volume). This diluted mixture was inoculated with activated Bacillus subtilis, initiating aerobic fermentation at 37°C for 1month. After fermentation, the supernatant was separated from the suspension by centrifugation, and the fermented garlic solution was concentrated using an evaporator until the NO2− concentration reached at least 300 ppm (calculated using the formula: 0.092 x measured value = 300 ppm). NO and SOD levels were measured using Hanna HI96707 meters (Hanna Instruments, Australia). For quality control, replicate samples and samples with known concentrations were analyzed.

In most cases, consumption of FGE does not lead to adverse events. However, if side effects do manifest, they are typically mild and may include diarrhea, stomach discomfort, bloating, heartburn, headache, heart palpitations, or nausea.

### 2.5 Medical device and pulse parameters

**Figure 1:**
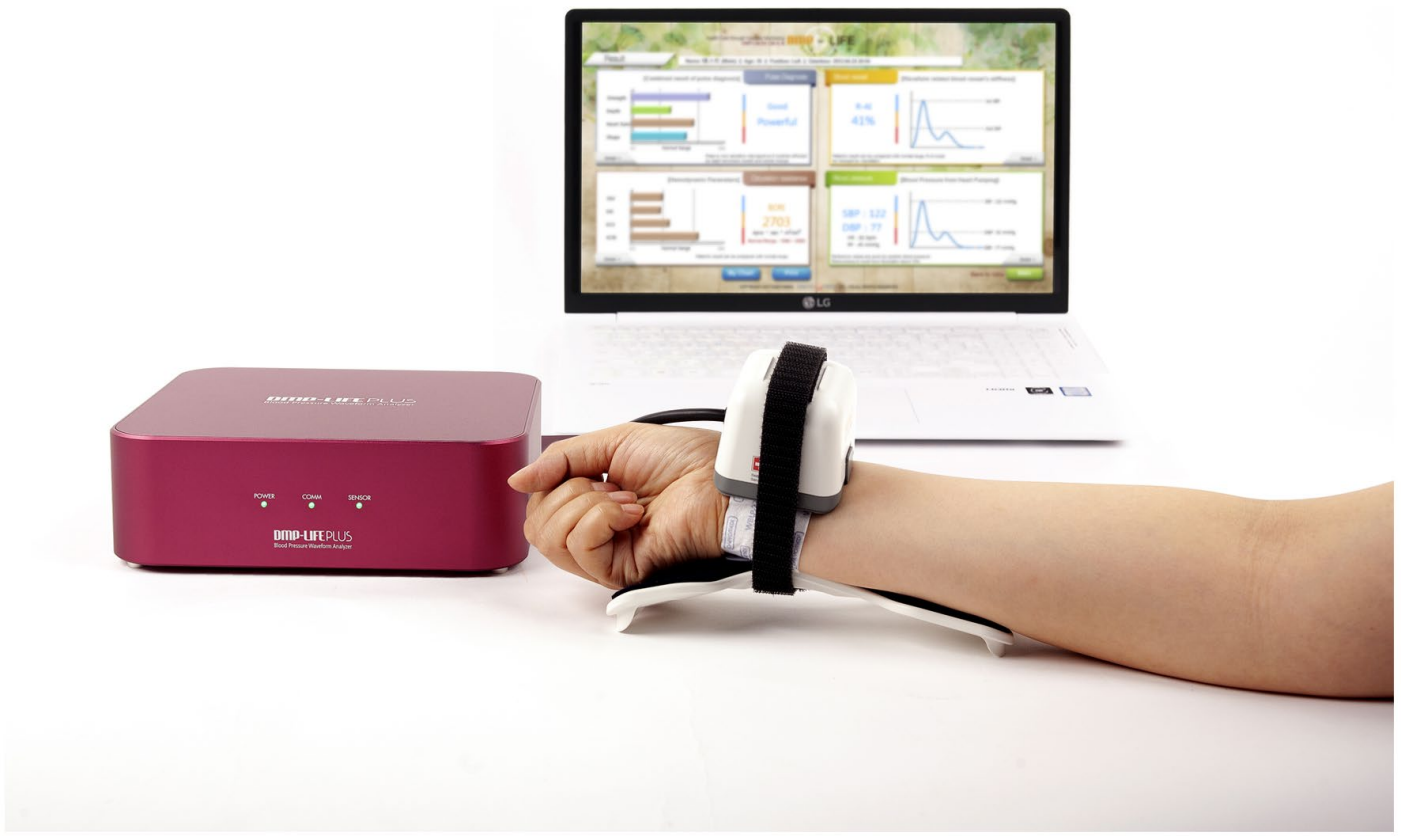
Noninvasive radial artery tonometry device (DMP-Lifeplus).

The DMP-Lifeplus, a commercially available noninvasive radial artery tonometry device developed by DAEYOMEDI Co., Ltd., in the Republic of Korea, was utilized in this study (Figure 1). The DMP-Lifeplus was manufactured in compliance with ISO 18615:2020—General requirements of electric radial pulse tonometric devices.

After positioning the semiconductor 5-channel-sensor array in a linear arrangement across the radial artery, the device measured the radial artery pulse waveform using a tonometric method.

The pulse parameters analyzed by the DMP-Lifeplus are summarized in Table 3.

**Table 3:** Pulse parameters and description.

Analysis variable (unit)	Description
Heart rate variability	
Low frequency (ms^2^)	Power of low-frequency component within 0.04–0.15 Hz
High frequency (ms^2^)	Power of high-frequency component within 0.15–0.4 Hz
LF/HF Ratio	Ratio of low-to high-frequency components
Pulse characteristic	
Mean applied pressure (kgf/cm^2^)	Calculated mean value of applied pressure during measurement
Radial augmentation index (%)	Calculated as h3/h1, a variable that evaluates the tension of peripheral blood vessels
Width (w, ms)	Time measured from the width of the two thirds of the first peak (H1)
Width/time (w/t, %)	Width (ms)/Time (t) ratio
Mean pulse width (kgf/cm^2^)	Pulse width of blood vessel when inflated
Stroke volume index (ml/m^2^)	The amount of blood ejected from the heart in one heartbeat, calculated as stroke volume divided by body surface area
Cardiac output index (L/min)	The amount of blood ejected from the heart in 1 min, calculated as cardiac output divided by body surface area

### 2.6 Statistical analysis

The normality of the distribution for each variable was verified using the Shapiro–Wilk test. Males and females at baseline were compared between groups using independent t tests and chi-square tests, respectively. Intergroup comparisons were performed using t tests or Mann–Whitney U tests, depending on whether the data was normally distributed. Repeated-measures analysis of variance (adjusted for sex, age and body mass index) was used to compare differences before and after the ingestion of FGE containing NO within the same group, not for between-group comparisons. While data on other potential confounders were collected, these variables were not included in the primary model to maintain simplicity and to account for sample size limitations, which could reduce statistical power when adjusting for multiple covariates.

Multiple imputation was used to address missing data. The SPSS Statistical Software Package (version 23.0; SPSS, Chicago, IL) was used for all analyses. The results were considered significant at *p* < 0.05.

## 3 Results

### 3.1 Participant characteristics

**Figure 2:**
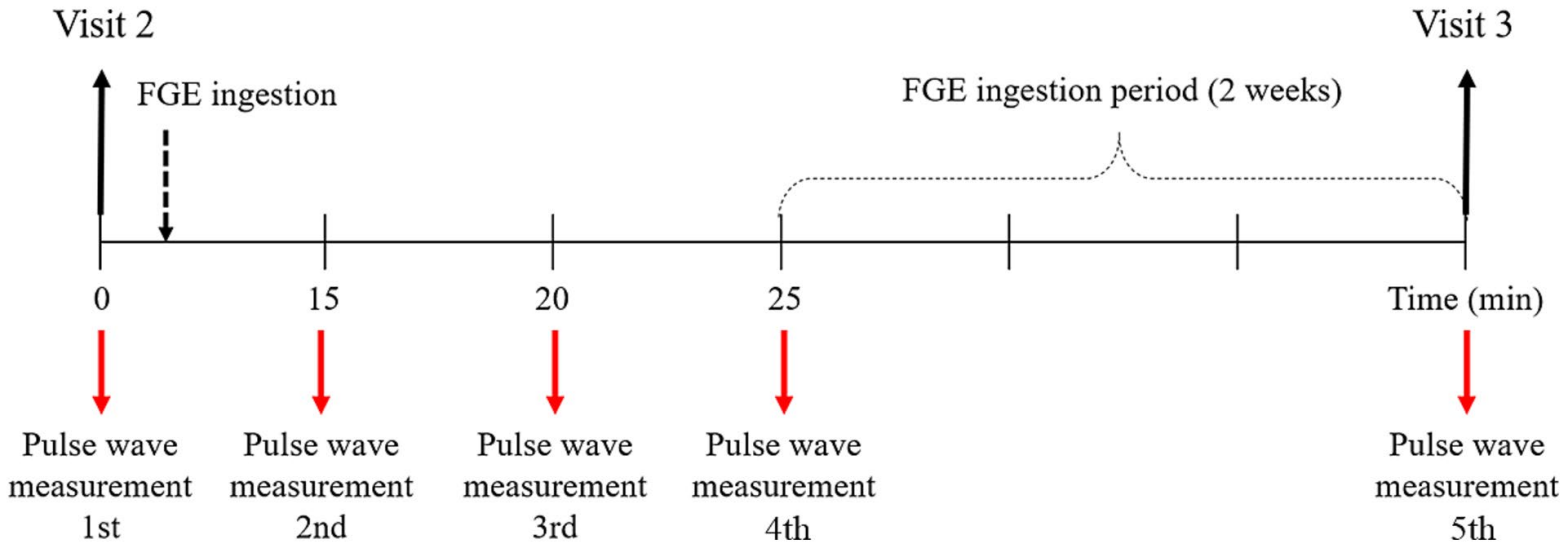
Flowchart of the study workflow.

One participant declined to participate; therefore, 40 participants were enrolled in this study. Ultimately, five participants were excluded from the analysis: one participant canceled the pulse wave evaluations, and four participants had missing data. Consequently, the dataset for analysis comprised 35 participants. Figure 2 provides an overview of the study workflow.

The characteristics of the participants (*n* = 35) in each group are summarized in Table 4. The compliance rate was determined to be approximately 89.8%.

**Table 4:** Participant characteristics (*N* = 35).

Variables	Male (*n* = 19)	Female (*n* = 16)	*p* value
Age (years)	53.16 (11.97)	56.19 (5.06)	0.326
Anthropometrics			
Height (cm)	171.85 (5.28)	157.99 (5.09)	0.000^†^
Weight (kg)	75.36 (12.71)	64.38 (14.27)	0.022^†^
Body mass index (BMI)	25.53 (4.25)	25.73 (5.00)	0.899
Vital signs			
Systolic blood pressure (mmHg)	137.68 (17.09)	123.43 (15.87)	0.016^†^
Diastolic blood pressure (mmHg)	88.211 (9.06)	83.50 (9.10)	0.136
Heart rate (beats per minute)	76.53 (9.34)	85.69 (12.52)	0.019^†^
Temperature (°C)	36.77 (0.51)	36.54 (0.71)	0.272

Values represent the mean (standard deviation). ^†^*p* < 0.05 between males and females.

The test of equal variances for the two sexes indicated no statistically significant difference in the age distribution between the two groups, confirming homogeneity (*F* = 0.083, *p* = 0.934). The mean ages of the male and female participants were 53.16 ± 11.97 years and 56.19 ± 5.06 years, respectively (*p* = 0.326). The height and weight differed significantly between the sexes, but body mass index (BMI) did not (*p* < 0.001, *p* = 0.022 and 0.899, respectively). Systolic BP significantly differed between the two groups (*p* = 0.016), whereas diastolic BP was not significantly different (*p* = 0.136). Heart rate significantly differed between the sexes (*p* = 0.019); however, no significant differences existed in temperature (*p* = 0.272).

### 3.2 Pulse wave variables

**Figure 3:**
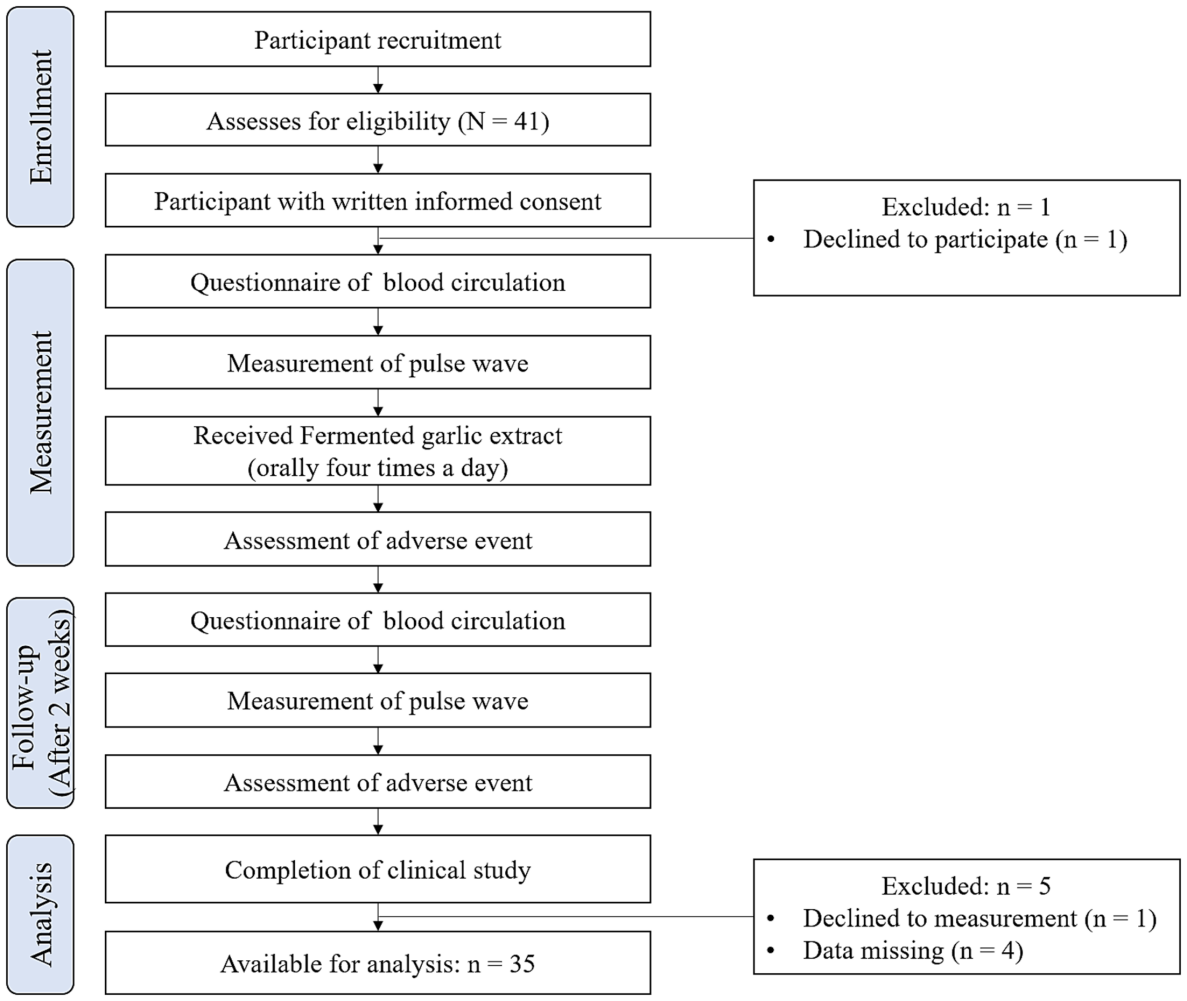
Pulse wave measurement timeline.

Before ingesting the FGE, the pulse waves of 35 participants were acquired using the DMP-Lifeplus. Then, the FGE was orally administered to the participants, and the pulse waves were measured at approximately 15, 20 and 25 min after ingestion and again after the 2-week ingestion period (Figure 3).

#### 3.2.1 Blood pressure and heart rate variability

Systolic and diastolic BP were significantly different between the measurements before and 15 min after ingestion of FGE containing NO but not after 2 weeks. No statistically significant differences were observed in heart rate variability, including LF, HF, or the LF/HF ratio, between before and after FGE ingestion (*p* > 0.05).

#### 3.2.2 Pulse characteristic parameters

Table 5 shows variations of pulse characteristic parameters according to ingestion times of 15, 20, and 25 minutes. The mean applied pressure (AP) significantly differed only at 20 min after ingestion (*p* = 0.025). There were significant differences in the RAI between before and 15 min after ingestion (*p* = 0.005) and between before and 25 min after ingestion (*p* = 0.001) but not after 20 min or 2 weeks (*p* = 0.077 and > 0.05). The w/t exhibited statistically significant differences between the measurements before ingestion and 15, 20, and 25 min after ingestion (*p* = 0.003 and *p* < 0.001, respectively) but not between before and 2 weeks after ingestion (*p* > 0.05). There were significant differences in w between before and 15, 20, and 25 min after ingestion (all *p* = 0.000) but not between before and 2 weeks after ingestion (*p* > 0.05). The mean pulse width (PW) differed significantly between before and 15, 20, and 25 min after (*p* = 0.005 and *p* < 0.001, respectively) but not between before and 2 weeks after (*p* > 0.05). There were no significant differences in the SVI between before and 15 and 25 min and 2 weeks after (all *p* > 0.05), whereas there were slight significant differences between before and 20 min after (*p* = 0.057). There were significant differences in COI between before and 20 and 25 min after (*p* = 0.003 and 0.012). Pulse characteristic parameters tended to decrease slightly during ingestion, except for the mean PW, which increased rapidly during ingestion.

**Table 5:** Comparison of the force axis parameters between baseline and after observation.

Variable	Pre	After 15 min	After 20 min	After 25 min	Post
Blood pressure					
Systolic BP (mmHg)	131.171	121.800^†^	-	-	127.657
Diastolic BP (mmHg)	86.057	79.486^†^	-	-	83.429
Heart rate variability					
LF	544.470 (67.510)	552.408 (43.147)	558.566 (45.911)	556.905 (49.520)	553.433 (49.753)
HF	494.291 (68.365)	502.527 (43.412)	509.124 (45.476)	506.553 (49.785)	503.964 (51.177)
LF/HF	1.105 (0.026)	1.100 (0.010)	1.098 (0.012)	1.100 (0.011)	1.099 (0.013)
Pulse characteristics					
Mean AP	178.199 (31.110)	170.599 (36.968)	164.952^†^ (29.292)	169.738 (37.955)	179.302 (51.026)
RAI	73.849 (12.206)	66.363^†^ (10.773)	67.837 (11.860)	63.814^†^ (13.045)	72.749 (15.570)
w/t ratio	0.281 (0.060)	0.252^†^ (0.062)	0.246^†^ (0.061)	0.239^†^ (0.063)	0.281 (0.069)
w	0.229 (0.032)	0.202^†^ (0.034)	0.201^†^ (0.041)	0.195^†^ (0.036)	0.225 (0.039)
Mean PW	16457.643 (4334.862)	19349.741^†^ (5422.833)	19337.993^†^ (4262.542)	20681.138^†^ (4804.539)	15944.616 (4106.728)
SVI	43.680 (7.747)	42.063 (7.112)	41.714 (7.541)	41.986 (7.810)	43.591 (7.499)
COI	3.111 (0.262)	3.046 (0.265)	2.971^†^ (0.303)	2.986^†^ (0.298)	3.157 (0.277)

Values represent the mean (standard deviation), ^†^*p* < 0.05 compared with values before and after FGE ingestion. Changes in pulse wave parameters from before FGE ingestion to 15, 20, and 25 min and 2 weeks after ingestion were analyzed by repeated-measures ANOVA. The participant’s age, sex, and body mass index were entered as covariates. The covariates were entered in a forward stepwise manner. Post hoc pairwise comparisons were performed with Bonferroni correction. BP, blood pressure; LF, low frequency; HF, high frequency; AP, applied pressure; RAI, radial augmentation index; w/t ratio, width/time ratio; w, width of pulse; PW, pulse width; SVI, stroke volume index; COI, cardiac output index.

### 3.3 Safety analysis

The National Cancer Institute (NCI) Common Terminology Criteria for Adverse Events (CTCAE) provides standardized grading for adverse events (AEs) related to drug treatment or medical devices, defining severity from mild (1) to death (5). This study used CTCAE v4.0 to assess safety in all participants who ingested the product at least once. No adverse events occurred during the study.

## 4 Discussion

This study hypothesized that 2 weeks of ingestion of FGE containing NO will improve cardiovascular health, including BP and arterial compliance. Contrary to this hypothesis, no significant changes were found after 2 weeks of FGE ingestion, but there was a significant change in the BP, mean AP, RAI, w, w/t ratio, SVI, COI, and mean PW immediately after FGE ingestion. The oral administration of low-dose FGE containing NO demonstrated acute positive effects on the wrist artery, reducing BP and improving arterial stiffness.

Similar to our previous research findings (13), we observed a consistent trend of decreased systolic and diastolic BP when comparing pre-and post-FGE ingestion. In a 2023 systematic review, Imaizumi et al. showed that the use of garlic can reduce BP and improve cardiovascular parameters such as carotid intima-media thickness (14). In a 2020 meta-analysis, Ried et al. suggested that garlic supplements significantly lowered systolic BP by an average of 8.3 ± 1.9 mmHg and diastolic BP by 5.5 ± 1.9 mmHg (15). Several studies have demonstrated that garlic can help lower BP in individuals with hypertension (16). A mean reduction of approximately 9 and 6 mmHg in systolic and diastolic BP, respectively, due to garlic supplementation can be expected in hypertension subjects (11). Similarly, in this study, systolic and diastolic BP decreased after FGE ingestion. The systolic and diastolic BP before FGE ingestion was 131.17 and 86.06 mmHg, respectively. Conversely, the BP decreased to 121.80 and 79.49 mmHg immediately after ingestion (a difference of 9.37 and 6.57 mmHg, respectively). The pattern of change before and after FGE ingestion showed a similar trend to that observed in previous studies.

The mean AP, RAI, w, w/t ratio, SVI, COI, and mean PW are representative indicators of arterial stiffness, and RAI, w, and w/t ratio tend to increase with age (17). A higher mean AP, RAI, w, w/t ratio, SVI, and COI typically indicate increased arterial stiffness, while lower values suggest greater arterial compliance or flexibility (18). The mean PW indicates the degree to which blood vessels dilate, reflecting their diameter and vasodilation. The more flexible the vessels are, the larger the mean PW becomes. Therefore, a larger mean PW indicates decreased arterial stiffness, while a smaller mean PW suggests decreased arterial compliance or flexibility.

The results of this study were similar to those of previous research across all pulse characteristics, with noteworthy findings observed in w, w/t, and mean PW. These variables exhibited significant changes at 15, 20, and 25 min after ingestion of FGE containing NO. Arterial stiffness and arterial compliance improved immediately after consuming FGE with NO. This finding suggests that FGE containing NO exerts an immediate effect on arterial stiffness, which is corroborated by the changes observed in pulse wave parameters. Szulińska et al. conducted a clinical study aimed at determining the effects of garlic extract on arterial stiffness. Obese patients were randomly assigned to receive garlic extract or placebo daily for 3 months, and arterial stiffness was reduced in the garlic extract-supplemented group (19). Another previous study investigated the effect of NO modulation on the RAI in patients with Turner syndrome and revealed that the administration of NO donors improved the decrease in the RAI, suggesting a relationship between NO and the RAI (20). Fitch et al. conducted a nonclinical study in rats to examine whether endogenous NO plays a role in the regulation of vascular stiffness. A bolus injection of a NO synthase inhibitor significantly increased the pulse wave velocity, accompanied by an increase in blood pressure. This additional increase in vascular stiffness may be due to remodeling of the vascular wall as a result of chronic NOS inhibition and hypertension (21). In contrast, Turner et al. reported conflicting results in their study of the effect of dried garlic powder on blood lipid levels, BP and arterial stiffness in a 12-week randomized controlled trial (RCT); however, garlic powder tablets had no clinically relevant BP or stiffness-lowering effects in middle-aged individuals (22).

NO ingestion causes vascular smooth muscle relaxation. The effects of nitrite concentrations on smooth muscle relaxation are attributed to their reduction to NO, which consequently activates soluble guanylyl cyclase in smooth muscle cells. Several previous studies have noted that nitrite concentrations above physiological levels activate soluble guanylyl cyclase and vasodilate vascular smooth muscle preparations, suggesting a role for nitrite in vasodilation (23, 24). In our study, the mean PW significantly differed at 15, 20, and 25 min after FGE ingestion, exhibiting a rapid increase. The results of this study are therefore consistent with the findings mentioned above.

Blood vessels undergo vasodilation in response to increases in blood flow (25). Parameters of arterial stiffness, such as pulse wave velocity, have recently been proposed as independent risk factors of cardiovascular events (26). Therefore, although we could not analyze indicators related to blood flow in our study, our results suggested that blood flow and pulse wave velocity would also improve.

Collectively, NO ingestion via low-dose FGE had acute positive effects on the wrist artery, and arterial function and cardiovascular physiology improved immediately after consuming an FGE containing NO.

This feasibility study has several limitations, including a small sample size, short ingestion period, and single-center clinical study design. Additionally, the single-arm study design limits the ability to compare outcomes with a control group, which restricts the generalizability of the findings. As a feasibility study, the sample size was determined based on practical considerations, such as study duration, budgetary constraints, and site-specific circumstances, rather than formal power calculations. This approach aligns with the exploratory nature of feasibility studies and recommendations from previous research. The limitations of the small sample size, short ingestion period, single-center setting, and single-arm design are acknowledged in the manuscript. Future follow-up studies with larger sample sizes, determined using formal statistical calculations, longer ingestion periods, and a randomized controlled trial design will be essential to test the hypothesis and validate these findings. Nevertheless, this study successfully assessed the effects of FGE containing NO using quantitative and objective pulse parameters as noninvasive indicators.

The effectiveness of FGE may be compromised in patients with various medical conditions and with age. Additionally, there are potential side effects and risks associated with the use of NO-related medications. Therefore, the use of NO-related treatments or supplements containing NO should be carefully monitored and prescribed by healthcare professionals based on individual health conditions. Consequently, we anticipate that the results of this study may serve as valuable guidance for the use of pulse wave parameters in the process of monitoring and prescribing treatments.

## Data Availability

The original contributions presented in the study are included in the article, further inquiries can be directed to the corresponding authors.
